# Prediction of Blood-Brain Barrier Penetration (BBBP) Based on Molecular Descriptors of the Free-Form and In-Blood-Form Datasets

**DOI:** 10.3390/molecules26247428

**Published:** 2021-12-07

**Authors:** Hiroshi Sakiyama, Motohisa Fukuda, Takashi Okuno

**Affiliations:** Department of Science, Faculty of Science, Yamagata University, 1-4-12 Kojirakawa, Yamagata 990-8560, Japan; fukuda@sci.kj.yamagata-u.ac.jp (M.F.); okuno@sci.kj.yamagata-u.ac.jp (T.O.)

**Keywords:** blood-brain barrier penetration (BBBP), free-form dataset, in-blood-form dataset, machine learning (ML), molecular descriptor, random forest (RF), forward search, deep neural network (DNN)

## Abstract

The blood-brain barrier (BBB) controls the entry of chemicals from the blood to the brain. Since brain drugs need to penetrate the BBB, rapid and reliable prediction of BBB penetration (BBBP) is helpful for drug development. In this study, free-form and in-blood-form datasets were prepared by modifying the original BBBP dataset, and the effects of the data modification were investigated. For each dataset, molecular descriptors were generated and used for BBBP prediction by machine learning (ML). For ML, the dataset was split into training, validation, and test data by the scaffold split algorithm MoleculeNet used. This creates an unbalanced split and makes the prediction difficult; however, we decided to use that algorithm to evaluate the predictive performance for unknown compounds dissimilar to existing ones. The highest prediction score was obtained by the random forest model using 212 descriptors from the free-form dataset, and this score was higher than the existing best score using the same split algorithm without using any external database. Furthermore, using a deep neural network, a comparable result was obtained with only 11 descriptors from the free-form dataset, and the resulting descriptors suggested the importance of recognizing the glucose-like characteristics in BBBP prediction.

## 1. Introduction

The blood–brain barrier (BBB) function of the brain capillaries protects the brain by suppressing the entry of chemicals from blood [1,2,3,4,5,6,7]. The BBB function is brought from the endothelial cells, that form the walls of the blood vessels, and the BBB regulates the movement of ions and molecules between the blood and the brain. In drug development, since brain drugs need to penetrate the BBB, the BBB permeability has been investigated in detail. On the other hand, a quick and reliable prediction of BBB penetration (BBBP) will be helpful for drug development [8,9]. Recently, a BBBP dataset for 2053 compounds were compiled and a computational prediction study was conducted using the molecular descriptors derived from the chemical structures [10]. This dataset has been adopted by MoleculeNet as a benchmark dataset for machine learning (ML) [11,12].

MoleculeNet recommended a methodology in predicting BBBP, especially for data split and evaluation metric. The most characteristic and important method is to use the fixed scaffold split. The procedure of this split is as follows: (1) the molecular compounds are grouped into scaffold sets on the basis of the skeletal ring structures termed scaffolds [13]; (2) the compounds are sorted in reverse order (descending sort), and the scaffold sets are sorted from the largest to the smallest; (3) the dataset are split into training, validation, and test data in an 8:1:1 ratio from the top. Then, this split remains fixed throughout the prediction. It should be noted that by sorting the scaffold sets from the largest to the smallest, the test data will consist of compounds that are less related to others. Since the scaffold split is unbalanced, the prediction model easily overfits to the training data; however, finding a good model under this difficult condition is valuable in predicting unknown data, because the actual unknown data that emerge in the future are not guaranteed to be similar to the existing data at all. Of course, the prediction score becomes better using the random split [14], but the fixed scaffold split, recommended by MoleculeNet, is still worth challenging.

As an evaluation metric for the BBBP prediction, MoleculeNet recommend the area under receiver operating characteristic curve (ROC-AUC) score [15]. The ROC-AUC metric is suitable for the BBBP prediction from the point of view of the brain–drug discovery, because the metric is probabilistic and sensitive to the prediction quality for both the positive and negative penetration. In the MoleculeNet paper [11], the best ROC-AUC score for the test data was 0.729 (KernelSVM). In the recent work, the score has been improved to 0.753 using the multichannel substructure-graph gated recurrent unit (MSGG) method by Wang and coworkers [16]. This score has been the highest value using the Moleculenet’s scaffold split algorithm without external training data. In this study, we aim to break the record.

The original BBBP dataset contains 2053 items with four attributes: the index number from 1 to 2053 (“num”), the name of the compound (“name”), the penetrating or non-penetrating properties (“p_np”), and the SMILES [17] string of the compound (“smiles”). The SMILES strings represent the chemical structures, and from the SMILES strings, useful features, termed molecular descriptors, are generated by programs, including RDKit [18] and Mordred [19]. The SMILES strings in the BBBP dataset are, however, usually based on the reported forms, and most of them are the as-isolated forms. That is, the form in blood or in permeating the BBB is not necessarily the same as the as-isolated form. In addition, the crystallographically observed structures are often used, and they are sometimes tautomers different from the commonly described formal chemical structures, and there are no perfect tools for converting these tautomers into common chemical structures. Therefore, in this study, the free-form and the in-blood-form datasets were manually prepared, by modifying the SMILES strings. The free form corresponds to the form in permeating the BBB by passive diffusion and is important especially for lipid soluble (hydrofobic) compounds. The in-blood form is especially important for some polar compounds, such as amino acids. The in-blood form may be related to the form that permeate the BBB by solute carriers [2].

**Figure 1:**
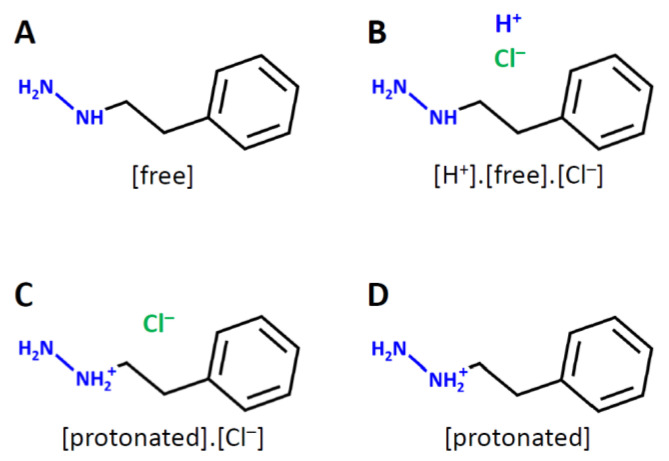
Chemical forms of an example compound: a free form (**A**), a frequently used formal salt form (**B**), an actual salt form (**C**), and a protonated form in aqueous solution (**D**).

Some compounds change their chemical forms depending on the environment. For example, amine compounds are not always easy to be isolated as their free forms (Figure 1A), and are often obtained as the hydrochloric salts (Figure 1B,C), for the isolation and purification purposes. The pH value of human blood stays between 7.35 and 7.45, and the aliphatic amines often exist as the protonated forms (Figure 1D) without counter anions. Another example is carboxylic acid. Sometimes carboxylic acids are isolated as their sodium salts, but they exist as the deprotonated forms in blood pH.

In addition, we have noticed the following problems in the SMILES strings in the BBBP dataset when obtaining the molecular descriptors: (1) when the drug is a mixture of two derivatives of the compound in a certain ratio, the SMILES string contains the both derivatives, and the calculated molecular weight, for example, will be about double of the single component; (2) when the compound contains crystal solvents or adducts, which are used for the isolation purpose in the synthetic procedure, molecular weights and so on will be different from those of the actual species; (3) when the SMILES string is simply wrong for some reasons, it cannot be a good datum. These cases are actually seen in the dataset and should be revised especially when the molecular descriptors are used. Furthermore, there are duplication and inconsistent problems as follows: (4) when the same compound appears twice or three times, it is not so preferable; (5) in addition, when the same compound appears twice and their penetration properties are different, the learning may be confused to some extent. In this study, we prepared the free-form and in-blood-form datasets, solving the above problems, and BBBP prediction was conducted by several ML methods.

## 2. Results and Discussion

### 2.1. Free-Form and In-Blood-Form Datasets

At the beginning of this study, two datasets (free-form and in-blood-form datasets) were manually curated by modifying the SMILES strings in the intact BBBP dataset. In the free-form dataset, the SMILES strings represent the single free neutral molecules, except for the quaternary ammonium cations. On the other hand, in the in-blood-form dataset, the SMILES strings represent the single dominant species expected at blood pH (~7.4). The intact dataset contains 2053 items, but after removing the duplicate and inconsistent items, the number of independent items became 1957 in the two new datasets. In the following comparison, only the corresponding 1957 items in the three datasets (intact, free-form, and in-blood-form datasets) are used.

**Figure 2:**
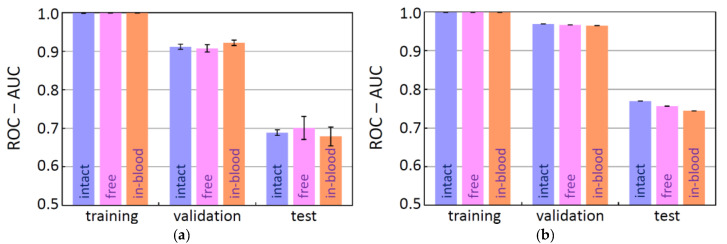
The ROC-AUC scores in comparison of the three datasets: intact, free-form, and in-blood-form datasets, using a DNN model (**a**) and a RF model (**b**) with 200 molecular descriptors.

With the intension of comparing the three datasets, 200 molecular descriptors were generated by RDKit from the SMILES strings of each dataset. (These 200 descriptors are so-called low-dimensional descriptors, which can be readily obtained without 3D structures.) Then, using the descriptors as input, the BBBP properties (positive (1) or negative (0)) were predicted by popular ML methods with the deep neural network (DNN) and the random forest (RF) models. For the data splitting, the fixed scaffold split method [11,12] was used to prepare training, validation, and test data. In this method, the split data were intentionally fixed to the same data throughout the whole procedure, as mentioned in the introduction. The resulting ROC-AUC scores are shown in Figure 2, where the scores are the averages of five trials (at the 100th epoch for the DNN results). The ROC-AUC scores for the validation and the test data fell in the ranges of 0.90–0.97 and 0.67–0.77, respectively, and the test scores were lower than the validation scores. This tendency is in concordant with that of the MoleculeNet results (validation: 0.94–0.97; test: 0.67–0.73) [11], indicating that the slight decrease in the number of items has little effect on the prediction tendency. Comparing the ROC-AUC scores for the test data (ROC-AUC(test)) in the DNN results, the free-form dataset was slightly better than the others. The ROC-AUC(test) scores for the RF results were slightly better than those for the DNN results, and no variance was observed in the RF scores (standard deviation = 0). The ROC-AUC(test) score of the intact dataset was the highest (0.770(0)); however, we do not consider the intact dataset anymore because it contains the inappropriate SMILES strings, as mentioned previously.

**Figure 3:**
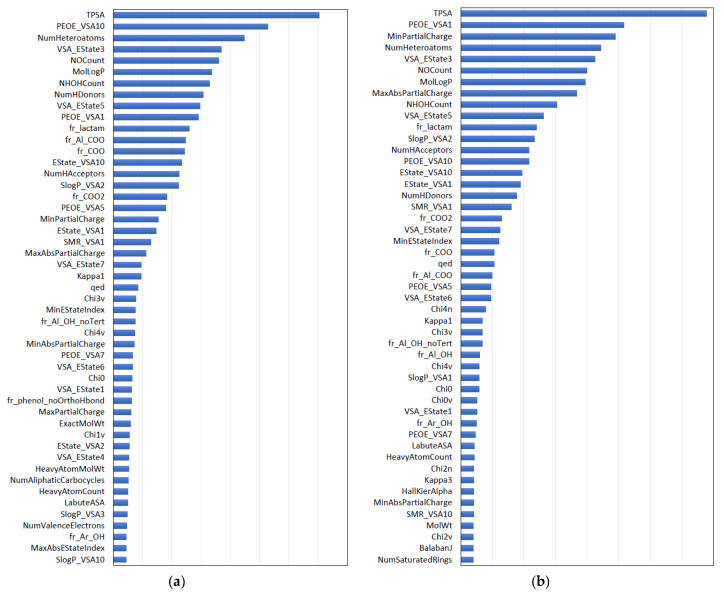
Importance of the top 50 molecular descriptors obtained by the RF method for the free-form dataset (**a**) and the in-blood-form dataset (**b**).

**Figure 4:**
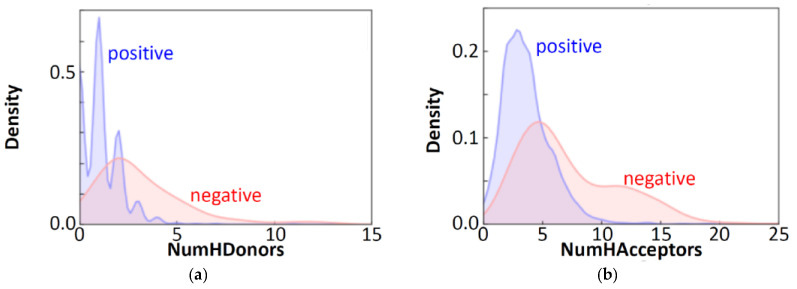

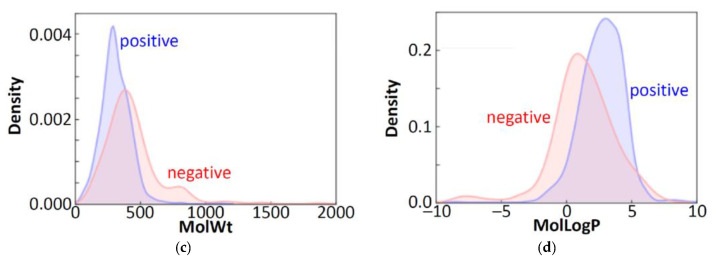
Distribution of positive and negative BBBP properties in the free-form dataset with respect to the number of hydrogen bond donors (**a**), the number of hydrogen bond acceptors (**b**), the molecular weight (**c**), and the MolLogP value (**d**).

The importance of the top 50 molecular descriptors, from the RF results, is shown in Figure 3 for the free-form and in-blood-form datasets. For both of the two datasets, nine descriptors with clear chemical meaning were found to be prominent, as summarized in Table 1. These descriptors are easy to interpret and immediately remind us of the factors in “the Lipinski’s rule of five” [20], for evaluating drug-likeness: H-bond donors, H-bond acceptors, molecular weight, and calculated LogP [21], although the molecular weight was less prominent in the results. The Lipinski’s rule is an empirical rule that relates the ease of absorption of orally administered drugs to the structures of the compounds; however, the rule was confirmed to be important also in the BBBP prediction of this study. According to the Lipinski’s rule, the drug absorption becomes poor when there are more than 5 H-bond donors, 10 H-bond acceptors, the molecular weight is greater than 500 and the calculated LogP is greater than 5. The BBBP showed the tendency to follow the Lipinski’s rule, as shown in Figure 4. It can be easily confirmed from the data, but we would like to emphasize that the important factors in ML prediction directly point us to the Lipinski’s rule.

What can be seen beyond the Lipinski’s rule is the lower limit in the MolLogP value (Figure 4d). That is, some compounds possessing the negative MolLogP values are permeable. The lipophilicity (or hydrophobicity), evaluated by LogP, is known to increase the permeability of lipophilic (or hydrophobic) compounds to some extent by passive diffusion [22], and the lipophilicity is one of the most important factors in BBBP [23,24]. However, the exceptional permeable hydrophilic compounds should be considered. We will discuss some of the related results in the next section.

### 2.2. Forward Search for Molecular Descriptor Sets by DNN

**Figure 5:**
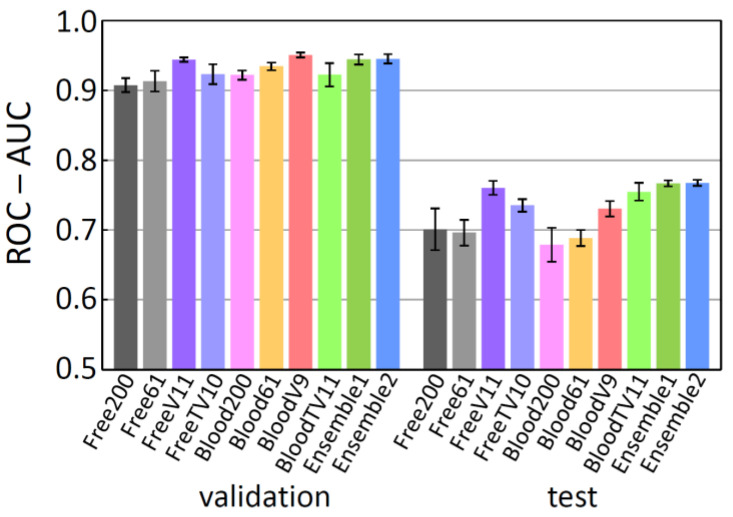
ROC-AUC scores of the prediction for the free-form and in-blood-form datasets obtained by DNN and ensemble methods.

To conclude this section in advance, in the following descriptor search process, the obtained descriptors suggest the importance of the glucose transporter in BBBP prediction, in addition to the importance of hydrogen bonds. The prediction score generally depends on the features as inputs. For the purpose of finding the efficient molecular descriptor set as features, the forward search was conducted by a DNN in the so-called low-dimensional descriptors, which can be readily obtained from the chemical structures of the compounds. The forward search method is suitable for finding good combinations of molecular descriptors. In this method, the most efficient descriptors are determined one by one on the basis of the ROC-AUC (validation) or the ROC-AUC (training + validation) score, until the score saturates. During this forward search the training data were used for training and the validation data for evaluation, in order to keep the test data uncontaminated for later fair model evaluations. The obtained four descriptor sets are included in Table 2, and the resulting ROC-AUC scores are shown in Figure 5. In each forward search, the ROC-AUC score almost converged when nine or ten descriptors had been found. The two descriptor sets, FreeV11 and FreeTV10 were obtained when the free-form dataset was used, and the other two, BloodV9 and BloodTV11, were obtained when the in-blood-form dataset was used. The characters “V” and “TV” stand for validation and “training + validation”, respectively, and indicate the criteria in choosing descriptors. The results with the 200 RDKit descriptors and the 61 RDKit descriptors (RDKit61 in Table 2) are also included in Figure 5, for the purpose of comparison. The 200 RDKit descriptors are all of the molecular descriptors that RDKit generates, and the 61 RDKit descriptors were obtained by filtering the 200 RDKit descriptors by removing the number-of-functional-group descriptors and the similar descriptors. For example, the “ExactMolWt” descriptor has been removed because it is similar to the “MolWt” descriptor.

The ROC-AUC(test) scores with the resulting forward-searched descriptor sets (0.730(11)–0.760(10)) were much higher than those with the 200 or 61 RDKit descriptors (0.68(2)–0.70(3)), and the score was found to vary greatly depending on the descriptors used. Among the single model results, the FreeV11 descriptor set with the free-form dataset showed the highest ROC-AUC(test) score (0.760(10)), and the BloodTV11 with the in-blood-form dataset the second-highest score (0.755(13)). If the average predicted probabilities between the highest and the second-highest results were calculated by an ensemble method, the score largely improved to 0.767(5) (Ensemble1 in Figure 5). When considering the *n*th power average, the smaller the *n*, the higher the ROC-AUC(validation) score, and the score almost converged when *n* = 1/32. In the ensemble method with the 1/32 power average, the ROC-AUC(test) score slightly improved to 0.767(4) (Ensemble2 in Figure 5).

In all the four forward-searched molecular descriptor sets, “the number of hydrogen bond donors (NumHDonors)” appeared, indicating the importance of hydrogen bonds in BBBP prediction. Other major descriptors are “the number of heteroatoms (NumHeteroatoms)” and “the number of oxygen atoms (nO)”, which are also related to the number of hydrogen bonds. These findings are consistent with the earlier reports claiming the importance of hydrogen bonds; that is, compounds forming more than six hydrogen bonds tend to have restricted entry into the central nervous system [2,23].

**Figure 6:**
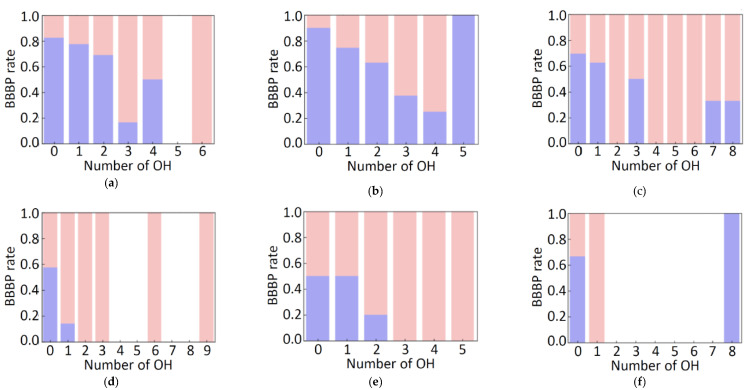
BBBP ratio with respect to the number of aliphatic heterocycles (*n*) and the number of aliphatic hydroxy groups excluding tertiary alcohol OH, showing BBBP positive (blue) and negative (pink) for *n* = 0 (**a**), 1 (**b**), 2 (**c**), 3 (**d**), 4 (**e**), and 5 (**f**).

**Figure 7:**
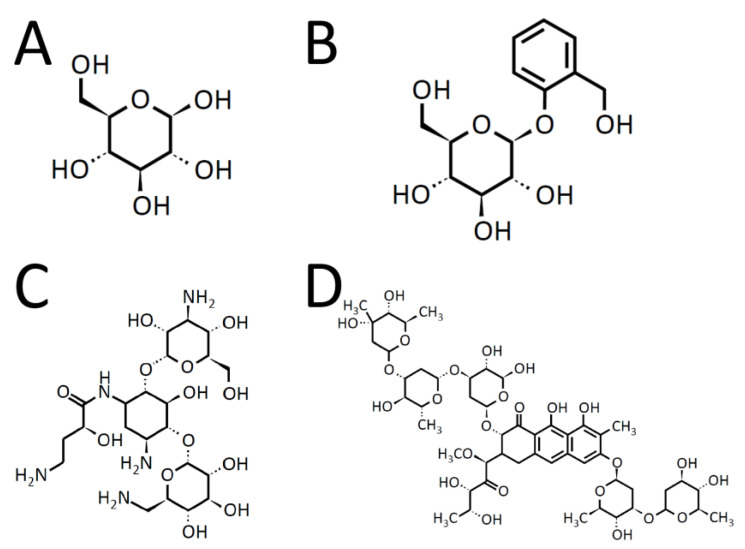
Chemical structures of β-glucose (**A**), salicin (**B**), amikacin (**C**), and plicamycin (**D**).

Another major descriptor is “number of aliphatic heterocycles (NumAliphaticHeterocycles)”, which is a new finding. The aliphatic heterocycles quickly remind us of glucose, which is the primary energy substrate of the brain [25]. Actually, in the descriptor set FreeV11, which gave the best score (ROC-AUC (test): 0.760(10)), most descriptors seem important for recognizing glucose. Yet another characteristic feature is “the number of aliphatic hydroxyl groups excluding *tert*-OH (fr_Al_OH_noTert)”, and this is also related to glucose. The BBBP ratio (penetrating and non-penetrating ratio) with respect to the number of aliphatic heterocycles and the number of aliphatic hydroxy groups is shown in Figure 6. In general, the larger the number of aliphatic hydroxy groups, the smaller the hydrofobicity, and the small hydrofobicity leads to the non-penetrating property. This tendency is observed also in Figure 6. However, there are some exceptions. The chemical structures of the typical three exceptions are depicted in Figure 7 with the chemical structure of β-glucose.

The exceptions (salicin, amikacin, and plicamycin) and β-glucose are all rich in hydroxy groups and hydrophilic; however, they all have the positive BBBP properties. The typical structural feature of the three exceptions is to have the glucose or glucose-derivative functional groups. The glucose transporter in BBB has been crystallographically characterized [26], and the transporter has been found to have a rather large cavity with dozen asparagine and glutamine residues. This suggests that the exceptions penetrate the BBB by being selectively incorporated in the glucose transporter.

Actually, 150 g of glucose are consumed per day in a brain [27]. Although glucose is hydrophilic, a large amount of glucose enters the brain using the glucose transporter. In the forward-search study using the DNN model, the permeability related to glucose was found by the combination of molecular descriptors, and the high ROC-AUC (test) score (0.760(10)) was achieved with only the eleven descriptors. Recognizing the permeability of the glucose-related compounds is of importance in distinguishing permeable hydrophilic compounds from the rest of the impermeable hydrophilic compounds, including mannitol.

### 2.3. BBBP Prediction by Some Other Models

**Figure 8:**
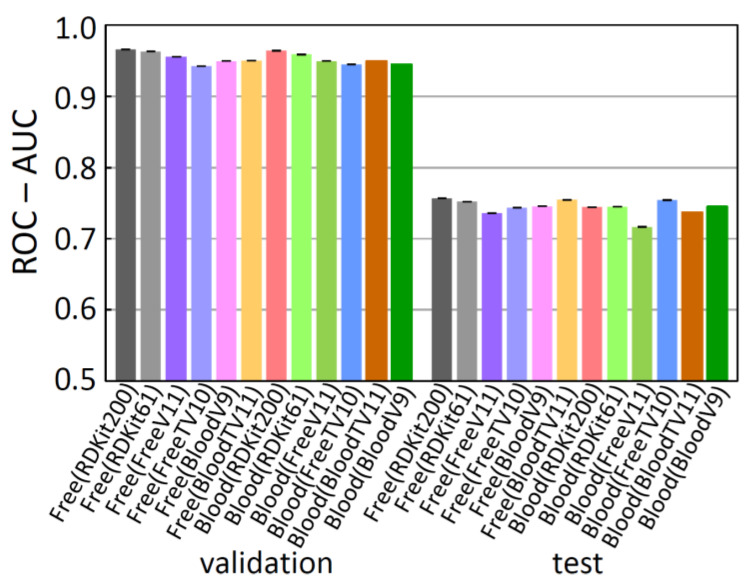
ROC-AUC scores of the prediction for the free-form and in-blood-form datasets obtained by RF method. Descriptor set is in parentheses.

Further RF-model prediction was conducted for the free-form and in-blood-form datasets by changing the set of molecular descriptors. The resulting ROC-AUC scores are depicted in Figure 8. Despite the improvement in the score by the descriptor selection for the DNN models, the scores for the RF models were not so improved with the sets of selected descriptors. On the other hand, for the RF model, the larger the number of descriptors, the higher the score seems to be. Furthermore, the addition of the twelve “Mordred” molecular descriptors gave the best ROC-AUC(test) score (0.773(0)) for the free-form dataset. The RF method was found to be fast and generally gave good scores without selecting descriptors. The CatBoost (CB) [28] method also gave good results without selecting descriptors, but the RF method was faster and better in the score.

### 2.4. Best Single Models and Their Ensemble

**Figure 9:**
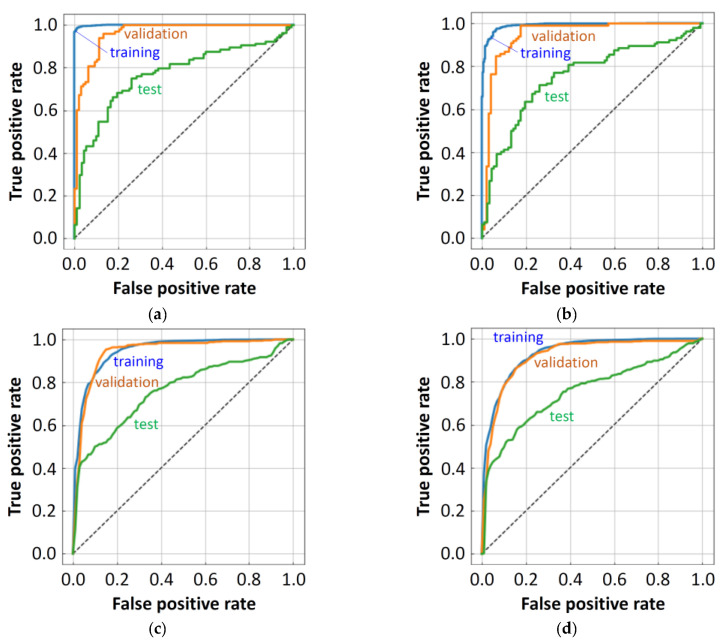
ROC curves for RF:Free(Large212) (**a**), CB:Free(Large212) (**b**), DNN:Free(FreeV11) (**c**), and DNN:Blood(BloodTV11) (**d**).

The top six single models with respect to the ROC-AUC (test) score are summarized in Table 3. The best score was obtained by the random forest (RF) model with Large212. The receiver operating characteristic (ROC) curves of this model are shown in Figure 9a. The training score was close to 1.00; the validation score was more than 0.96; and the test score was 0.773(0). The order of these scores was reasonable; however, the model might have slightly overfitted to the training data. Similar ROC curves were also obtained for other RF results. A result by the CB model, ranked 5th, was somewhat similar to those of the RF models. A good result was obtained with a large descriptor set, and the tendency of the ROC curves (Figure 9b) was also similar to that of the RF models. For the DNN models (Figure 9c,d), on the contrary, the obtained ROC curves for the training and the validation were similar to each other in each case (free-form dataset ranked 3rd and in-blood-form dataset ranked 6th). The DNN results were not so good with large descriptor sets, but descriptor selection was quite efficient in improving the score. The 3rd ranked DNN result was obtained, using only the eleven descriptors, while the 1st and 2nd RF results needed more than 200 descriptors.

**Figure 10:**
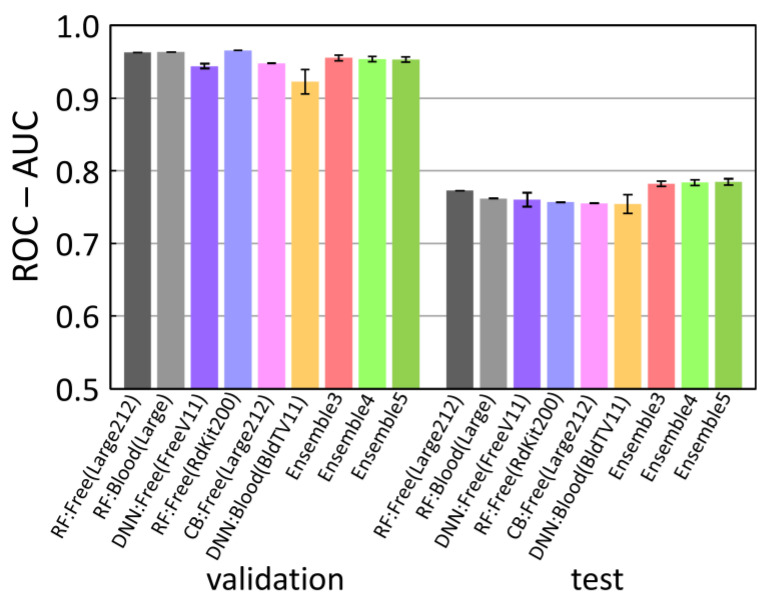
ROC-AUC scores of the top six prediction results and ensemble results.

Ensembles of the single-model results often give better results. As far as we have tried, the best combination is the 1st RF(Free-form), 3rd DNN(Free-form), and the 6th DNN(In-blood-form) results, which are included in Figure 10 together with the single model results. In the equally weighted simple average (Ensemble3), the ROC-AUC (test) score was improved to 0.782(4). In the 1/32 power average, the ROC-AUC (test) score was improved to 0.784(4) (Ensemble4). Furthermore, if the clipping was introduced, modifying the probabilities less than 0.02 to 0 and the probabilities larger than 0.98 to 1, the score was improved to 0.785(4) (Ensemble5).

### 2.5. Comparing the Predictive Ability with Other Works

**Figure 11:**
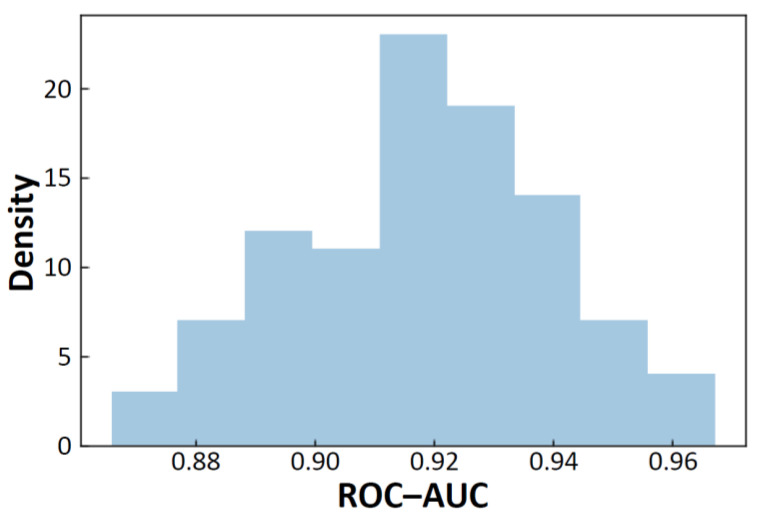
Distribution of ROC-AUC scores for RF:Free(Large212) with scaffold split.

Both the best single model score (0.773(0)) and the best ensemble score (0.785(4)) in our study were higher than the existing best score (0.753 [16]) using the Moleculenet’s fixed scaffold split, without using any external database for training. In order to confirm the excellence of our model, the confidence interval was estimated for the best single model, using 100 scaffold-split sets (Figure 11). (Note that these scaffold-split sets are different from that of MoleculeNet. See Section 3.5.) The 95% confidence interval was obtained as (0.914, 0.923), and its lower limit value was much larger than the earlier best score (0.753). This indicates that the best single model in our study gave really better score than the earlier model. In addition, this confidence interval investigation indicated that the MoleculeNet’s scaffold split is really a difficult task. That is, the score using the MoleculeNet’s scaffold split (0.773(0)) is well below the confidence interval and looks like an outlier. The difficulty is primarily due to the order of the original dataset [10] and is caused by the reverse sorting in the MoleculeNet’s algorithm. Such a difficulty may be appropriate for finding a good model. However, it is important to know that the scaffold split of the BBBP dataset with the MoleculeNet’s algorithm is much more difficult and gives a much lower score than other scaffold splits. If there is a model that can give a good score using the very difficult MoleculeNet’s algorithm, it will be a model with really great predictive ability. On the other hand, if we use random split for the “RF:Free(Large212)” model, for instance, the 95% confidence interval of the ROC-AUC score becomes (0.927, 0.935), and this is better than that using the scaffold split. The recently reported high-scoring results were not based on the MoleculeNet’s scaffold split [14,29,30,31] or with using the external biological database [32].

**Figure 12:**
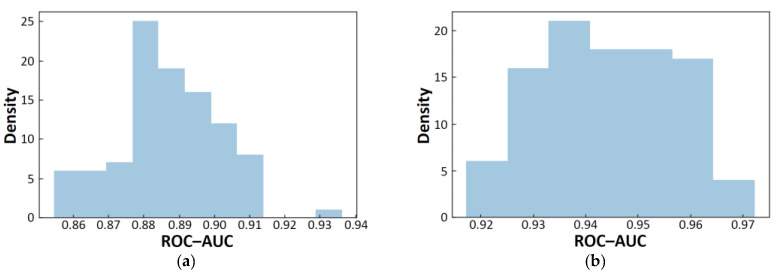
Distributions of ROC-AUC scores for the the test set of 31 compounds (**a**) and for the the test set of 95 compounds (**b**).

Here we compare our best single model result with the results of the two most recently published papers [30,31]. Shakel and coworkers used 10-fold cross-validation to achieve the ROC-AUC score of 0.93 [30]. Then, they evaluated their model using an external test set of 74 compounds to achieve the ROC-AUC score of 0.90. On the other hand, Liu and coworkers achieved the ROC-AUC score of 0.957 using 5-fold cross-validation and the ROC-AUC score of 0.966 with their ensemble model [31]. They evaluated their ensemble model using an external test set of 213 compounds to achieve the ROC-AUC score of 0.834. Of the 74 items in the first external test set [30], 70 items were readable by RDKit; among them, after removing duplication with the MoleculeNet’s BBBP dataset, 31 items were unique. The ROC-AUC score in reference [30] was 0.90, while our 95% confidence interval of the ROC-AUC score was (0.884, 0.890) for the 31 items. Since the ROC-AUC score in reference [30] is included in our distribution (Figure 12a), the results can be said to be comparable. On the other hand, of the 213 items in the second test set [31], 204 were readable, and after removing duplication with the MoleculeNet’s BBBP, 95 unique items were obtained. The ROC-AUC score in reference [31] was 0.834 for the external test set, while our 95% confidence interval was (0.941, 0.946) for the 95 items (Figure 12b). Since our 95% confidence interval is higher than the ROC-AUC score in reference [31], our result seems to be better. In this study, the incorrect data were manually corrected in advance. This may be one of the reasons for the good score.

The ROC-AUC score of our best single model was 0.773(0) using the MoleculeNet’s scaffold split, and this score looks inferior to the scores of 0.93 and 0.966 in the recently reported k-fold cross-variation and ensemble results [30,31]. However, by comparing the ROC-AUC scores using the two external test sets, our single-model result was found to be comparable or better than the recently reported results. This may indicate that the MoleculeNet’s scaffold split algorithm is useful for finding good models. The score is strongly dependent on the test set, and if the training set contains data similar to the test set, the resulting score will be very high. We should be aware of this fact and pay close attention to the evaluation of the models. Once a new type of compound is adopted as an effective therapeutic agent, a group of similar compounds will be a new test set. In such a case, it is not possible to include similar data in the training set in advance. Machine learning is good at interpolation. However, the population of drug candidates may change in the future and extrapolation may be required. The MoleculeNet’s scaffold split seems to assume this extrapolation to some extent. From this point of view, it is worth using the scaffold split algorithm proposed by MoleculeNet.

### 2.6. Comparing the Results of Free-Form and In-Blood-Form Datasets

When comparing the free-form dataset and the in-blood-form dataset, the free-form dataset was found to give slightly better results in this study. This seems to be consistent with that the passive diffusion is dominant in BBB permeability and that lipophilicity (hydrophobicity) is an important factor for BBB permeability [22,23,24]. For hydrophilic compounds, the in-blood form seems to be more appropriate, but in fact it turns out to be better represented by free-form descriptors. The idea of the most populated neutral tautomer at pH 7.0 was tried earlier [33], but using the free form was found to be better in our present study.

## 3. Materials and Methods

### 3.1. Computations

All the computations were conducted with Python (3.7.9) [34] under Anaconda (2020.02) [35] and Jupyterlab (1.2.6) [36] environment on a Windows 10 computer with Intel Core i7-10510U CPU, Inspiron 7391 (Dell, Round Rock, TX, USA).

### 3.2. Dataset and Preprocessing

The BBBP dataset, provided on the MoleculeNet website [12], is based on the original 2053 data compiled by Falcao and coworkers [10]. The BBBP dataset contains 2050 items, presumably because the three duplicate data have been removed (num = ‘63’ (=‘73’), ‘1086’ (=‘1741’), and ‘1161’ (=‘435’)). (Note: “num” is the identification number in the dataset). The original 2053 data are provided as the supporting information of reference [10].

In the first preprocessing stage of the BBBP dataset, the three missing data were added from the original data. In addition, since eleven of the data (num = ‘60’, ‘62’, ‘393’, ‘616’, ‘644’, ‘647’, ‘648’, ‘649’, ‘650’, ‘651’, ‘687’) cannot be handled by RDKit (2019.09.3.0) [18], their SMILES strings were modified to be processed by RDKit. In this paper, this dataset is called intact dataset. This dataset is deposited as an electronic file named “bbbp_intact.csv” (Supplementary Materials).

In the second preprocessing stage, adducts, biproducts, and counter ions were manually removed from the SMILES strings, because some molecular descriptors cannot be correctly obtained with the adducts and so on. At the same time, some mistaken structures were corrected (e.g., num = ‘653’ and ‘1506’), according to the original literature [37]. Then functional groups of the compounds were changed to their free forms to provide neutral molecules, except for quaternary ammonium cations. (Here, “free form” means a chemical form that is not ionized and is electrically neutral.) Furthermore, duplication and inconsistent data were removed to obtain 1957 independent data. The duplication removed here also includes the duplication of the same main components with different counter ions. Removed items by duplication are summarized in Table 4. In the twelve duplicate pairs, the identical items were found to show different BBBP properties, and these inconsistent pairs were removed from the dataset as shown in Table 4. In this paper, the obtained dataset is called free-form dataset, which is deposited as an electronic file named “bbbp_free.csv”.

In the third preprocessing stage, deprotonation and protonation were taken into account at blood pH (7.35–7.45) on the basis of the general pKa values of the functional groups and the total charge. In this process, the SMILES strings in the free-form dataset were manually modified one by one. (Actually, H.S. worked 1–2 h a day and took 2 months). In this paper, the resulting dataset is called in-blood-form dataset, which is deposited as an electronic file named “bbbp_blood.csv”.

The external test sets [30,31] were obtained from the “LightBBB” web site [38] and the supporting information of reference [31]. The datasets were used after removing duplications with the MoleculeNet BBBP data. The resulting datasets are deposited as electronic files nemed “lbbb31.csv” and “tx95.csv”.

### 3.3. Molecular Descriptors

The molecular descriptors were generated by two programs, RDKit [18] and Mordred [19]. Since the descriptor ‘Ipc’ contains extremely large numbers, the descriptor values were divided by 1 × 10^41^. In the forward search by DNN, the descriptors were divided by each maximum value.

### 3.4. Models

The models used in this study were as follows: a DNN model with four layers, a random forest (RF) model, and a CatBoost (CB, 0.24.1) [28] model. The DNN model was built with TensorFlow (2.1.0) [39] and Keras (2.3.1) [40]; each hidden layer consisted of 256 units. The activation function was the scaled exponential linear unit (SELU) [41] in the first three layers and sigmoid in the last layer. The model optimizer was AdaMax [42], and the loss function was binary cross entropy. The batch size was 32. The RF model was introduced from scikit-learn [43]; the number of trees in the forest was 90 and the maximum depth of the tree was 10. The hyperparameters were manually tuned at an early stage in this study and were used unchanged throughout this study. The tuning was done on the basis of the ROC-AUC value for the validation data.

### 3.5. Confidence Interval

The 100 scaffold-split sets, used for calculating the confidence intervals, were obtained as follows. As the result of the MoleculeNet’s scaffold split, the numbers of training, validation, and test data were 1565, 196, and 196, respectively. Of the 1565 training data, 1179 belonged to the scaffold/non-scaffold groups with two or more elements, and the remaining 386 belonged to the scaffold groups containing only a single element. Both the 196 validation data and the 196 test data belonged to the scaffold groups containing only a single element. The 778 (=386 + 196 + 196) data, belonging to the single-element groups, were randomly split into 386:196:196, and the 386 data were used as training data along with the 1179 data belonging to the multi-element groups. The remaining two 196 datasets were used as validation and test sets, respectively. By repeating the above series of operations 100 times, the 100 scaffold-split sets were prepared. The confidence intervals were calculated by SciPy [44].

## 4. Conclusions

In this study, aiming for quick and reliable blood–brain-barrier penetration (BBBP) prediction, machine learning (ML) was conducted, using readily prepared molecular descriptors. Since the actual unknown compounds that will appear in the future are not al-ways similar to the existing compounds, we intentionally used unbalanced data split, generated by the MoleculeNet’s scaffold split algorithm.

At the beginning, the molecular structures in the provided BBBP dataset were modified to create free-form and in-blood-form datasets. In this process, data defects were fixed, mistakes were corrected, and duplicates were removed, reducing the number of the data to 1957. Up to 212 molecular descriptors were generated by RDKit and Mordred for each molecular structure and used as inputs for ML. Each dataset was split using the scaffold split algorithm MoleculeNet used, and the split was maintained throughout the learning and prediction processes. 

In the BBBP prediction by several ML methods, the random forest (RF) model performed well (ROC-AUC: 0.773 with the 212 descriptors from the free-form dataset), and the obtained score was better than the existing best scores (0.753 [16]). The obtained important descriptors suggested the importance of the Lipinski’s rule in predicting BBBP. For the RF model, the larger the number of descriptors, the higher the score, and the RF method was found to be fast and gave good scores without selecting descriptors. Using the MoleculeNet’s scaffold-split set, the ROC-AUC score was 0.773, but using 100 scaffold-split sets for comparison yielded the ROC-AUC score of 0.918 with the 95% confidence interval of (0.914, 0.923). It turned out that the model can predict the dataset with high accuracy, and outperformed all other models on this benchmark.

On the other hand, a deep neural network (DNN) was found to be suitable for the forward search of molecular descriptors. As a result, forward search using DNN gave comparable predictive performance with only 11 descriptors (ROC-AUC: 0.760(10)), indicating the importance of the proper combination of molecular descriptors. The combination of the obtained descriptors suggested the importance of recognizing glucose-like characteristics, suitable for the glucose transporter.

Comparing the free-form dataset with the in-blood-form dataset, the free-form dataset was found to give slightly better results. This seems to be consistent with that the passive diffusion is dominant in BBBP, indicating the importance of considering the molecular descriptors for the free form in predicting BBBP.

Finally, the created datasets in this study are expected to be useful in future studies.

## Figures and Tables

**Table 1:** Nine descriptors with clear chemical meaning obtained by the random forest method.

Molecular Descriptor	Meaning ^1^
NumHeteroatoms	the number of heteroatoms
NOCount	the number of nitrogen and oxygen atoms
MolLogP	Wildman-Crippen LogP value [21]
NHOHCount	the number of NH and OH bonds
NumHDonors	the number of hydrogen bond donors
fr_lactam	the number of β-lactams
NumHAcceptors	the number of hydrogen bond acceptors
fr_COO2 and fr_COO	the number of carboxylic acids
fr_Al_OH_noTert	the number of aliphatic hydroxyl groups excluding *tert*-OH

^1^ The description is based on the RDKit documentation [18].

**Table 2:** Set of molecular descriptors.

Name of Descriptor Set	Molecular Descriptors
FreeV11	NumHeteroatoms, NumHDonors, NHOHCount, NumHAcceptors, NumSaturatedHeterocycles, fr_Al_OH_noTert, NumAliphaticHeterocycles, nH, NOCount, qed, nO
FreeTV10	NumHDonors, NumSaturatedHeterocycles, nO, NumAliphaticRings, MolWt, MolLogP, nN, fr_Al_OH, fr_SH, fr_ketone
BloodV9	NumHeteroatoms, MaxAbsPartialCharge, NOCount, NumHDonors, NumAliphaticHeterocycles, nS, fr_C_S, fr_unbrch_alkane, fr_ester
BloodTV11	NOCount, MaxAbsPartialCharge, NumHDonors, NumAliphaticHeterocycles, nO, MolWt, NumHeteroatoms, qed, NumHAcceptors, HeavyAtomCount, nN
RDKit61 (Free61, Blood61)	MaxEStateIndex, MinEStateIndex, MinAbsEStateIndex, qed, MolWt, MinPartialCharge, MaxAbsPartialCharge, FpDensityMorgan1, BalabanJ, BertzCT, Chi0, HallKierAlpha, LabuteASA, PEOE_VSA1, PEOE_VSA10, PEOE_VSA11, PEOE_VSA12, PEOE_VSA13, PEOE_VSA14, PEOE_VSA2, PEOE_VSA3, PEOE_VSA4, PEOE_VSA5, PEOE_VSA6, PEOE_VSA7, PEOE_VSA8, PEOE_VSA9, SMR_VSA1, SMR_VSA10, SMR_VSA2, SMR_VSA3, SMR_VSA4, SMR_VSA5, SMR_VSA6, SMR_VSA7, SMR_VSA9, TPSA, EState_VSA1, EState_VSA10, EState_VSA11, EState_VSA2, EState_VSA3, EState_VSA4, EState_VSA5, EState_VSA6, EState_VSA7, EState_VSA8, EState_VSA9, VSA_EState1, VSA_EState10, VSA_EState2, VSA_EState3, VSA_EState4, VSA_EState5, VSA_EState6, VSA_EState7, VSA_EState8, VSA_EState9, FractionCSP3, MolLogP, MolMR
RDKit200	200 RDKit descriptors [18]
Large	RDKit200 + {nH, nC, nN, nO, nS, nP, nF, nCl, nBr, nI, nX} from Mordred [19]
Large212	RDKit200 + {nH, nB, nC, nN, nO, nS, nP, nF, nCl, nBr, nI, nX} from Mordred [19]

**Table 3:** Top six single models.

No	Method	Dataset	Descriptor Set	ROC-AUC(Training)	ROC-AUC(Validation)	ROC-AUC(Test)
1	RF	Free-form	Large212	0.999(0)	0.963(0)	0.773(0)
2	RF	In-blood-form	Large	0.999(0)	0.964(0)	0.762(0)
3	DNN	Free-form	FreeV11	0.948(4)	0.944(3)	0.760(10)
4	RF	Free-form	RdKit200	0.999(0)	0.966(0)	0.757(0)
5	CB	Free-form	Large212	0.990(0)	0.948(0)	0.755(0)
6	DNN	In-blood-form	BloodTV11	0.934(14)	0.923(17)	0.755(13)

**Table 4:** List of removed 93 items.

Category	Removed Items
One of each two-identical-compound set (60 sets, 60 items)	‘63’ (=‘73’), ‘154’ (=‘129’), ‘312’ (=‘97’), ‘337’ (=‘29’), ‘384’ (=‘62’), ‘388’ (=‘96’), ‘394’ (=‘70’), ‘415’ (=‘3’), ‘422’ (=‘105’), ‘435’ (=‘13’), ‘453’ (=‘87’), ‘457’ (=‘52’), ‘468’ (=‘467’), ‘488’ (=‘46’), ‘489’ (=‘56’), ‘508’ (=‘50’), ‘533’ (=‘34’), ‘535’ (=‘75’), ‘562’ (=‘140’), ‘591’ (=‘72’), ‘593’ (=‘2’), ‘607’ (=‘490’), ‘616’ (=‘62’), ‘619’ (=‘567’), ‘644’ (=‘26’), ‘646’ (=‘60’), ‘649’ (=‘40’), ‘650’ (=‘58’), ‘651’ (=‘393’), ‘667’ (=‘4’), ‘668’ (=‘33’), ‘669’ (=‘93’), ‘670’ (=‘89’), ‘671’ (=‘69’), ‘672’ (=‘79’), ‘673’ (=‘23’), ‘690’ (=‘55’), ‘959’ (=‘450’), ‘966’ (=‘691’), ‘1073’ (=‘523’), ‘1085’ (=‘564’), ‘1086’ (=‘1741’), ‘1111’ (=‘555’), ‘1388’ (=‘296’), ‘1462’ (=‘139’), ‘1471’ (=‘585’), ‘1508’ (=‘191’), ‘1567’ (=‘344’), ‘1583’ (=‘654’), ‘1597’ (=‘219’), ‘1662’ (=‘437’), ‘1782’ (=‘45’), ‘1882’ (=‘700’), ‘1906’ (=‘15’), ‘1947’ (=‘101’), ‘1961’ (=‘142’), ‘1971’ (=‘664’), ‘1979’ (=‘189’), ‘2031’ (=‘245’), and ‘2045’ (=‘252’)
Two of each three-identical-compound set (6 sets, 12 items)	‘269’ (=‘83’ =‘569’), ‘435’ (=‘13’ =‘1161’), ‘534’ (=‘85’ =‘1541’), ‘565’ (=‘59’ =‘617’), ‘566’ (=‘49’ =‘618’), ‘569’ (=‘269’ =‘569’), ‘617’ (=‘59’ =‘565’), ‘618’ (=‘49’ =‘566’), ‘713’ (=‘315’ =‘1916’), ‘1161’ (=‘13’ =‘435’), ‘1541’ (=‘85’ =‘534’), and ‘1916’ (=‘315’ =‘713’)
Inconsistent pair (12 pairs, 24 items) (Note: The number “1” represents penetrating, and “0” non-penetrating properties.)	(‘1’ [1], ‘380’ [0]), (‘17’ [1], ‘552’ [0]), (‘53’ [1], ‘648’ [0]), (‘102’ [0], ‘1009’ [1]), (‘128’ [0], ‘1701’ [1]), (‘176’ [0], ‘1645’ [1]), (‘267’ [0], ‘1314’ [1]), (‘284’ [0], ‘1881’ [1]), (‘305’ [0], ‘1361’ [1]), (‘325’ [0], ‘1910’ [1]), (‘326’ [0], ‘1381’ [1]), and (‘571’ [0], ‘1338’ [1])

## Data Availability

All the data created in this study are deposited as Supplementary Materials: “bbbp_intact.csv”, “bbbp_free.csv”, and “bbbp_blood.csv”.

## Supplementary Materials

The following are available online. Electronic files named “bbbp_intact.csv”, “bbbp_free.csv”, “bbbp_blood.csv”, “lbbb31.csv”, “tx95.csv”.

## Sample Availability

Samples of the compounds are not available from the authors.
